# Bio-Optics Based Sensation Imaging for Breast Tumor Detection Using Tissue Characterization

**DOI:** 10.3390/s150306306

**Published:** 2015-03-16

**Authors:** Jong-Ha Lee, Yoon Nyun Kim, Hee-Jun Park

**Affiliations:** 1 Department of Biomedical Engineering, School of Medicine, Keimyung University, 1095, Dalgubeol-daero, Daegu 704-701, Korea; E-Mail: hjpark@kmu.ac.kr; 2 Department of Internal Medicine, Dongsan Medical Center, Keimyung University, 1095, Dalgubeol-daero, Daegu 704-701, Korea; E-Mail: ynkim@dsmc.or.kr

**Keywords:** tumor detection, artificial palpation, lesion characterization, tactile sensor, biomimetic sensor, Young’s modulus

## Abstract

The tissue inclusion parameter estimation method is proposed to measure the stiffness as well as geometric parameters. The estimation is performed based on the tactile data obtained at the surface of the tissue using an optical tactile sensation imaging system (TSIS). A forward algorithm is designed to comprehensively predict the tactile data based on the mechanical properties of tissue inclusion using finite element modeling (FEM). This forward information is used to develop an inversion algorithm that will be used to extract the size, depth, and Young's modulus of a tissue inclusion from the tactile data. We utilize the artificial neural network (ANN) for the inversion algorithm. The proposed estimation method was validated by a realistic tissue phantom with stiff inclusions. The experimental results showed that the proposed estimation method can measure the size, depth, and Young's modulus of a tissue inclusion with 0.58%, 3.82%, and 2.51% relative errors, respectively. The obtained results prove that the proposed method has potential to become a useful screening and diagnostic method for breast cancer.

## 1. Introduction

During the past two decade, various methods have been devised for measuring or estimating the soft tissue elasticity [1]. Generally this is called elasticity imaging. Elasticity imaging is currently performed using magnetic resonance elastography (MRE), atomic force microscopy (AFM), and ultrasound imaging. MRE is a dynamic approach where external oscillation is applied to the material and acoustic strain waves caused by the oscillation are visualized [2]. AFM has also been utilized to detect tissue elasticity; however, this is for small local area measurements [3]. Both MRE and AFM are extremely expensive and cumbersome techniques. Ultrasound is perhaps the most intensely investigated area for elasticity imaging [4,5]. Ultrasound can be divided into three cases: elastography, transient elastography, and sonoelastography. In elastography, compression is applied to the tissue then pre-compression and post-compression echo return signals are compared using correlation techniques to calculate the strain map in the tissue [6,7,8]. Transient elastography uses a low frequency transient vibration to create displacements in tissue, which are then detected using pulse-echo ultrasound [9]. Sonoelastography uses real-time ultrasound Doppler techniques to image the vibration pattern resulting from the propagation of low frequency shear waves that are propagated through the tissue [10,11,12]. The research on elasticity imaging shows that elastic modulus information has the potential to distinguish between malignant and benign tumors [13,14,15]. It is difficult to compare each technology because both technologies are at their infancy, however, from the physics point of view, ultrasound will suffer from limited contrast and resolution problems. 

Breast nodule stiffness is an acknowledged indicator of breast health, with increased tissue stiffness pointing to an increased risk of breast cancer [16]. Palpation of the breast to determine breast stiffness is an established screening method for assessing breast health. Women are advised to undergo a clinical breast examination (CBE) every year [17]. At present, physicians write up their findings, which are accompanied by a hand drawing of the breast mass. Thus, it is difficult to quantify the tactile sensation presented by the breast tumor. The efficacy of CBE is also limited by the experience of the physician. A noninvasive method for recording and estimating the stiffness would offer great clinical utility. The stiffness can be quantified using Young’s modulus. In addition to increased tissue stiffness, geometric parameters such as size and depth of the stiff region are important factors in assessing the tumor. The combined knowledge of tumor stiffness and geometry would aid tumor identification and help physicians select an appropriate treatment strategy. The objective of this research is to develop a methodology to estimate the mechanical properties of an embedded tumor based on the data obtained by the tactile sensation imaging system (TSIS). We proposed the design concept for the TSIS and tactile data processing methodology used to quantify various parameters—size, depth, and Young’s modulus—of an embedded tumor with a geometry representative of breast pathology. For this purpose, finite element method (FEM) and neural network (NN) algorithm are utilized. FEM is used to generate simulated tactile data on the tissue surface over different embedded tumor parameters in the idealized breast model. We employ a NN algorithm to map the simulated tactile data to the tumor parameters. We then compare the NN training results with those obtained from a realistic tissue phantom. The proposed characterization method was validated by the realistic tissue phantom with inclusions to emulate the tumors. The phantom was made of a silicone composite having a Young’s modulus of approximately 5 kPa. The inclusion was made using another silicone composite; the stiffness of which was higher than the surrounding tissue phantom. The Young’s modulus of each inclusion was 120 kPa, which is for fibrous tissue at 5% pre-compression with loading frequency of 4.0 Hz. 

## 2. Sensor Design and Sensing Principle

In this section, we present the design concept of the artificial palpation sensor in detail.

### 2.1. Sensor Design

**Figure 1:**
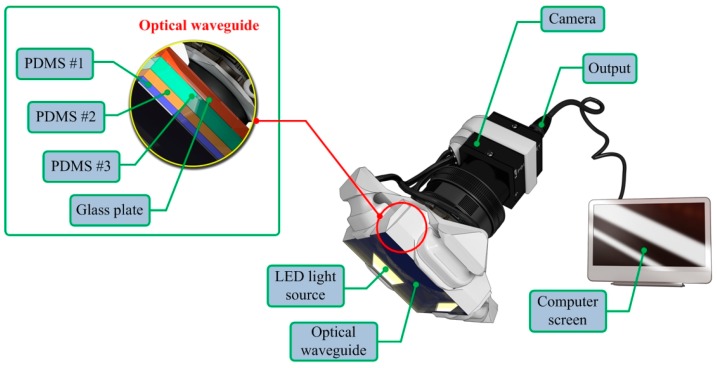
Schematic of the TSIS.

The TSIS incorporates an optical waveguide unit, a light source unit, a high resolution camera unit, and a computer unit. The optical waveguide is the system’s main sensing probe. The waveguide is composed of polydimethylsiloxane (PDMS), which is a high-performance silicone elastomer. In the current design, the waveguide needs to be flexible and transparent, and PDMS meets this requirement. To achieve the level of sensation of human touch, we emulated the tissue structure of the human finger. The human finger tissue is composed of three layers with different elastic moduli, specifically the epidermis, dermis, and subcutaneous layer. The epidermis is the hardest layer, with the smallest elastic modulus, and it is approximately 1 mm thick. The dermis is a softer layer, and it is approximately 1 to 3 mm thick. The subcutaneous is the softest layer and fills the space between the dermis and bone. It is mainly composed of fat and functions as a cushion when a load is applied to the surface. Due to the difference in hardness of each layer, the inner layer deforms more than the outmost layer when the finger presses into an object. To emulate this structure, three PDMS layers with different elastic moduli were stacked together. PDMS layer 1 is the hardest layer, the PDMS layer 2 is the layer with medium hardness, and the PDMS layer 3 is the layer with the least hardness. The height of each layer is approximately 2 mm for PDMS layer 1, 3 mm for PDMS layer 2, and 5 mm for PDMS layer 3. Figure 1 shows the schematic of the proposed sensor.

The high resolution camera used was a mono-cooled complementary camera with an individual pixel size of 4.65 μm (H) × 4.65 μm (V). The maximum lens resolution was 1392 (H) × 1042 (V) with an angle of view of 60°. The camera was placed below an optical waveguide. A heat-resistant borosilicate glass plate was placed between the camera and the waveguide to sustain an optical waveguide without losing the camera resolution. The internal light source was a micro-LED with a diameter of 3 mm. There were four LED light sources placed on four sides of the waveguide to provide sufficient illumination. The direction and incident angle of light were calibrated to be totally reflected in the waveguide. 

### 2.2. Sensing Principle

**Figure 2:**
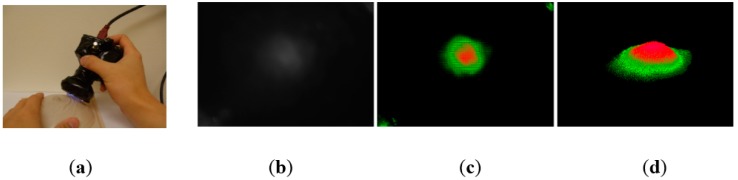
(**a**) Tactile sensation imaging system and breast phantom; (**b**) Obtaining tactile image of a tissue inclusion (Raw gray-scale tactile image); (**c**) Color visualization; (**d**) 3-D reconstruction.

**Figure 3:**
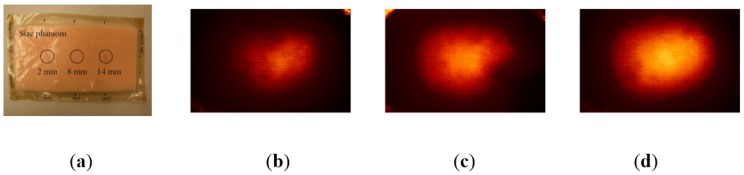
(**a**) Tissue phantom with different size of inclusions; (**b**) The tactile image of 2 mm size inclusion; (**c**) 8 mm size inclusion; (**d**) 14 mm size inclusion.

The proposed TSIS operates on the principle of total internal reflection (TIR). According to Snell’s law, if two mediums have different refractive indices, and the light is shone through those two mediums, then a fraction of light is transmitted and the rest is reflected. If the incident angle is above the critical angle, then TIR occurs. In the current system design, since the waveguide is surrounded by the air, and has a lower refractive index than PDMS layers, the incident light directed into the waveguide is totally reflected in the waveguide. The waveguide is transparent and flexible. Consequently, if a waveguide is compressed by an external force towards a stiff inclusion, the contact area of the waveguide deforms and causes the light to scatter. The scattered light is then captured by the high-resolution camera and saved as an image. Thus, the basic principle of tactile sensation imaging lies in capturing of the light scattered due to the inclusion. Figure 2 shows the TSIS and obtained tactile image. Figure 3 shows the tactile image obtained from the tissue phantom with different size of inclusions. 

## 3. Tactile Data Processing Algorithm

**Figure 4:**
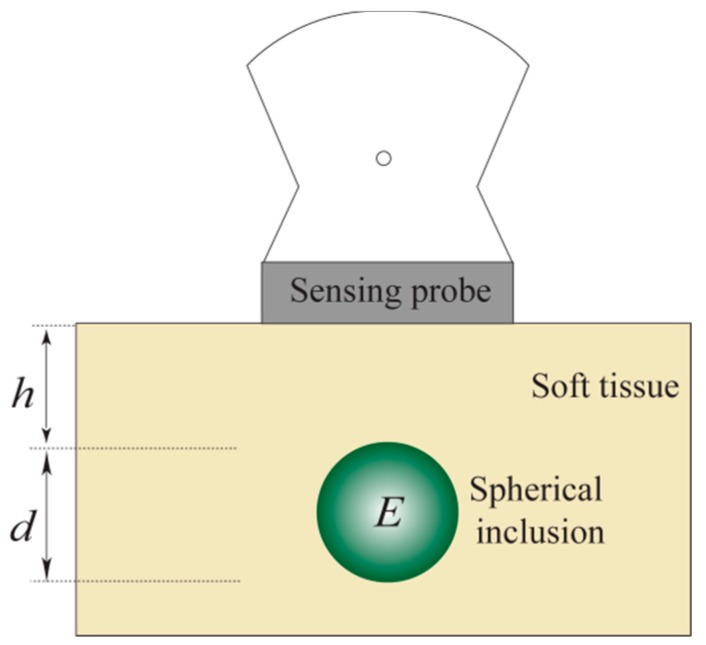
The half section of an idealized model for estimating the tumor parameters. The tumor diameter is d , the depth is h , and the Young’s modulus is E .

The idealized model used to estimate the various tumor parameters derived from the tactile data is shown in Figure 4. The assumptions used in the proposed idealized model are as follows.

(1) Most breast tumors are found in the upper outer quadrant of the breast where the tissue is relatively thin and flat [18]. Therefore, in our model, the tissue is approximated as a slab of material of constant thickness that is fixed to a flat, incompressible chest wall.
(2) The inclusion is assumed to be spherical and stiffer than the surrounding tissue. Wellman *et al.* used a breast modeling algorithm to investigate this assumption [19,20]. In clinical tests, they found that the results matched well with their breast modeling results under this assumption.
(3) We assume that both the tissue and the inclusion are linear and isotropic. Glandular and adipose tissues, which account for most of the breast tissue, are well modeled by isotropic materials [21].
(4) In this model, the indentation is made by the sensing probe of the TSIS with finite length. The interaction between the sensing probe and the tissue is assumed to be frictionless.

In the next section, we devise a methodology for estimating three parameters of the embedded inclusion: size (*d*), depth (*h*) and Young’s modulus (*E*). The estimation of these parameters will be extracted from the TSIS tactile data obtained at the tissue surface.

### 3.1. Forward Modeling–Finite Element Method 

**Figure 5:**
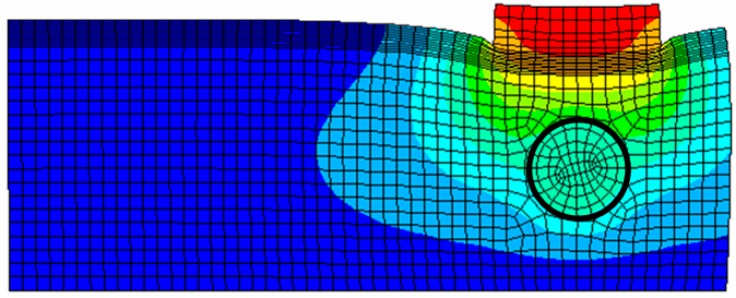
FEM model of an 8 mm diameter inclusion embedded in tissue 20 mm thick. The inclusion is located 5 mm beneath the tissue surface. The sensing probe of the TSIS is also shown in top of the tissue model.

We use finite element method (FEM) to investigate the tactile data obtained by the TSIS on the surface of the tissue with embedded inclusion. Figure 5 presents a graphical representation of the FEM model. The FEM model consists of tissue, inclusion, and sensing probe of TSIS. The FEM model comprises 3000 finite elements. The FEM is performed by the following assumptions [22,23].

(1) The biological tissue and inclusion are elastic and isotropic. It means the properties of a material are identical in all directions.
(2) The Poisson’s ratio of each material is set to 0.49 because the breast can be considered an incompressible material. The incompressible material means the material incapable of or resistant to compression.
(3) The biological tissue is assumed to be sitting on non-deformable hard surfaces like bones.
(4) The tissue cross-section is a square with dimensions 120 mm × 120 mm. The sensing probe of the TSIS is a square shape with dimensions of 25 mm × 25 mm, which corresponds to the sensing probe size in the laboratory design discussed in Section 2.

All are modeled using SOLID95 3 D elements available in ANSYS. Appropriate surface-to-surface contact elements have been defined in the ANSYS database model. The cross-section FEM meshed model with tissue, inclusion, and sensing probe of the TSIS is shown in Figure 5.

### 3.2. Tactile Data from FEM 

**Figure 6:**
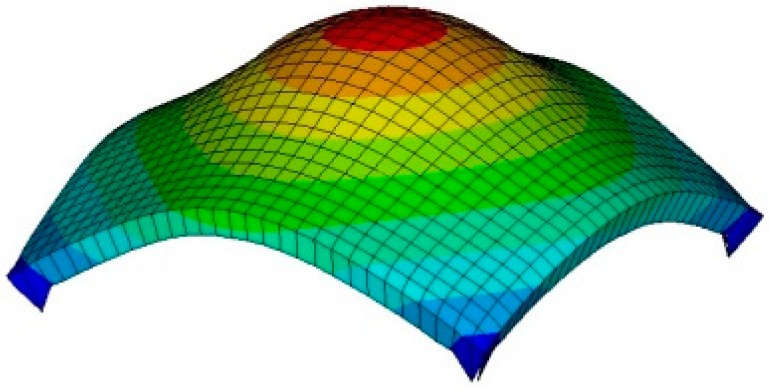
FEM result of sensing probe deformation. The result was obtained when the sensing probe was compressed against tissue containing a stiff inclusion embedded in tissue 20 mm thick. The inclusion was 5 mm diameter and it was located 5 mm beneath the tissue surface.

If the sensing probe compresses against the tissue containing a stiff inclusion, the deformation occurs. In the FEM, we capture the deformed shape in response to different parameters of inclusions. Figure 6 represents the sensing probe deformation captured by FEM. To quantify the FEM tactile data, we use the following three definitions: maximum deformation *d_total_*, total deformation *d_area_*, and deformation area *d_probe_* of sensing probe. The maximum deformation *d_total_* is defined as the maximum displacement of elements from the bottom surface of the sensing probe. The unit of the maximum deformation is *mm*. The total deformation *d_area_* is defined as the displacement summation of elements from the bottom surface of the sensing probe. The unit of the total deformation is *mm*. The deformation area *d_probe_* is defined as the deformed bottom surface area of the sensing probe where the displacement of finite elements is greater than 0. The unit of the deformation area is *mm^2^*.

**Figure 7:**
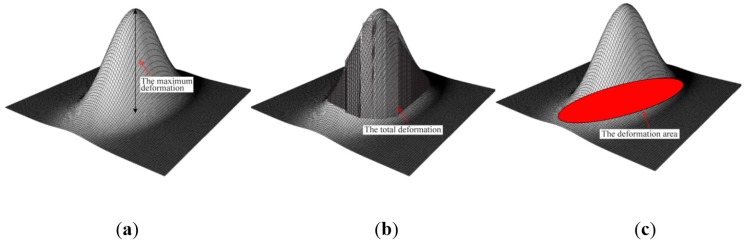
The quantification of FEM tactile data. (**a**) The maximum deformation; (**b**) The total deformation; and (**c**) The deformation area of sensing probe.

**Figure 8:**
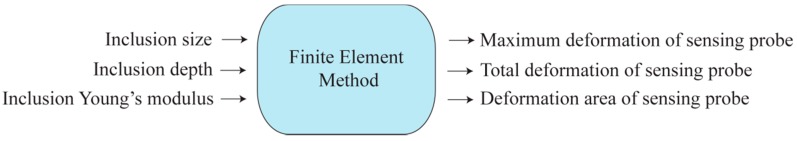
The inclusion size, depth, and Young’s modulus are the variables of FEM. The outputs of FEM are the maximum deformation, total deformation, and deformation area of sensing probe.

**Figure 9:**
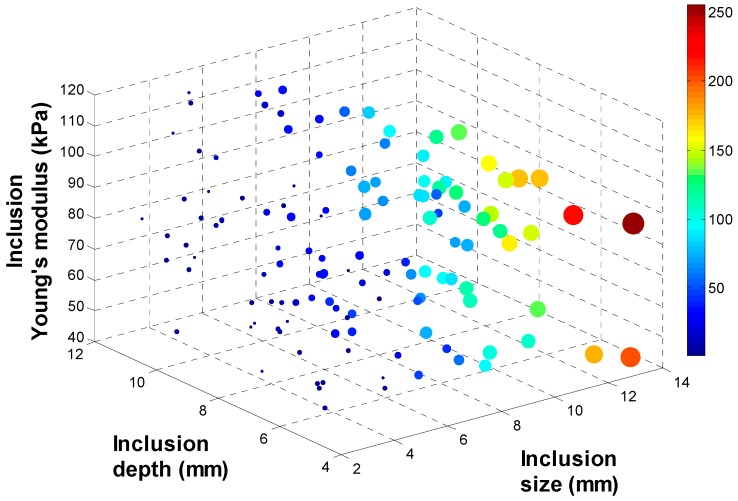
The maximum deformation of sensing probe depending on the inclusion size, depth, and Young’s modulus. The 4 D color dimension shows the maximum deformation value.

**Figure 10:**
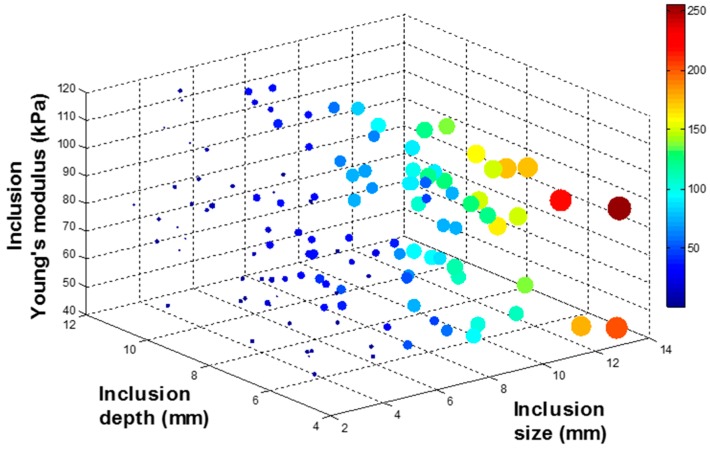
The total deformation of sensing probe depending on the inclusion size, depth, and Young’s modulus. The 4 D color dimension shows the maximum deformation value.

To investigate the relationship between (*d*, *h*, *E*) and (*d_total_*, *d_area_*, *d_probe_*), 134 input data sets are randomly generated as inputs of FEM. Among 134 input data sets, 9 input data sets are used to build the calibration tissue phantom. 134 output data sets corresponding to 134 input data sets are being generated through FEM. To visualize 134 data sets in 3 D space, output data sets are rescaled to [0:255] and displayed as colored circles at the locations specified by three inputs (*d*, *h*, *E*). The area of each marker is determined by the values in the vector and the colors of each marker are based on the values from 0 to 255. Figure 7, Figure 8, Figure 9 and Figure 10 represent the FEM tactile data with respect to changing (*d*, *h*, *E*). From the results, we notice that as the size of inclusion increases, the tactile data increases as the effect of bigger inclusion will cause more change in the sensing probe deformation. As the depth of inclusion increases, the tactile data decreases as the effect of stiff inclusion gets reduced and sensing probe presses the soft tissue. As the Young’s modulus of inclusion increases, the tactile data increases as the stiff inclusion makes the sensing probe to deform more.

The diagram of input and output of FEM is shown in Figure 8.

### 3.3. Tactile Data from TSIS 

**Figure 11:**
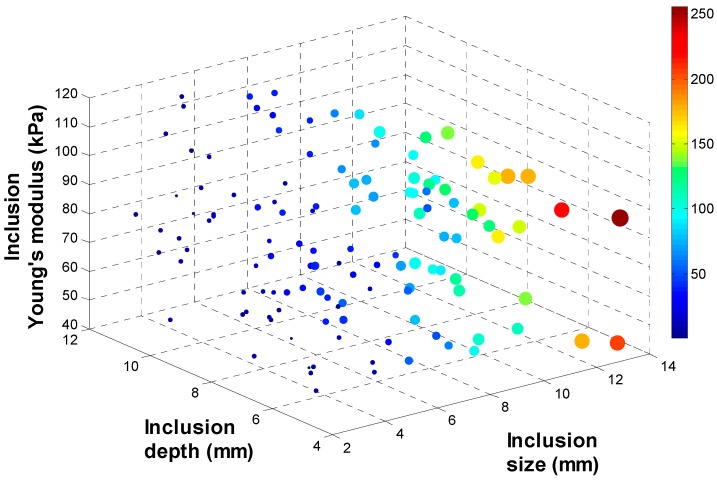
The deformation area of sensing probe depending on the inclusion size, depth, and Young’s modulus. The 4 D color dimension shows the maximum deformation value.

As we discussed in previous section, TSIS captures the dispersed light due to the sensing probe deformation and save it as an image format. To quantify the TSIS tactile data, we measure the maximum pixel value $O_{TSIS}^{1}$, the total pixel value $O_{TSIS}^{2}$, and the deformation area of pixel $O_{TSIS}^{3}$ in the tactile sensation image. The maximum pixel value $O_{TSIS}^{1}$ is defined as the pixel value in the centroid of the image. The range of the maximum pixel value is from 0 to 255. The total pixel value $O_{TSIS}^{2}$ is defined as the summation of pixel value in the image. The range of the total pixel value is from 0 to 255 × 1024 × 768. The deformation area of pixel $O_{TSIS}^{3}$ is defined as the number of pixel greater than the threshold value k in the image. In the current study, we set k as 5. Figure 11 represents the deformation area of sensing probe depending on the inclusion size, depth, and Young’s modulus.

### 3.4. Calibration: Mapping TSIS Tactile Data to FEM Tactile Data

**Figure 12:**
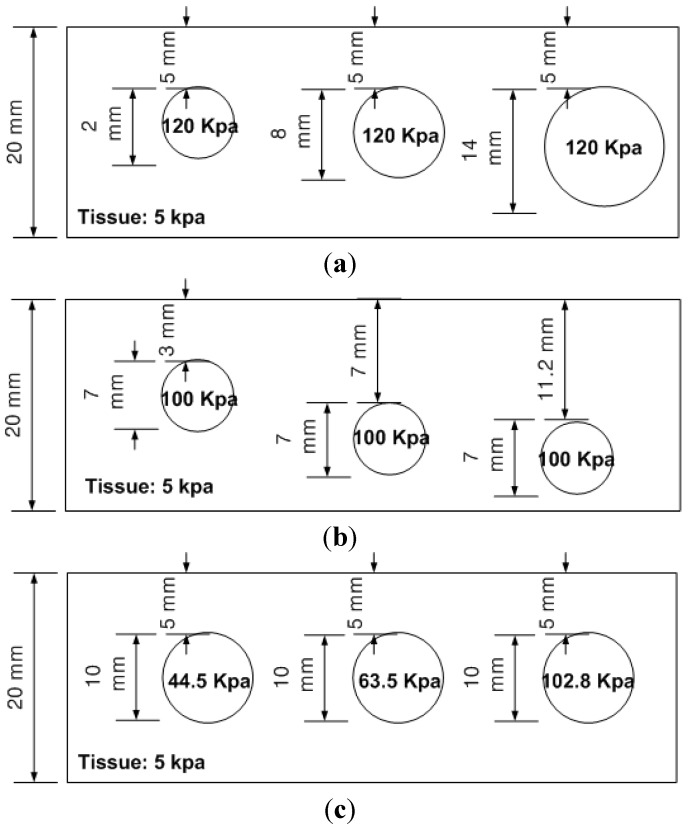
The schematic of calibration phantoms. The calibration phantoms are (**a**) Size phantom; (**b**) Depth phantom and (**c**) Hardness phantom. Each phantom includes 9 inclusions.

Since the FEM measures the quantity of the sensing probe deformation and the TSIS measures the quantity of the dispersed light due to the sensing probe deformation, it is necessary to relate the FEM tactile data and TSIS tactile data. This is the calibration process. For this purpose, we obtained tactile data of calibration phantom using both FEM and TSIS. The calibration phantoms consist of three phantoms, size, depth, and hardness phantoms. Each phantom contains three inclusions, resulting in a total of nine inclusions. Every inclusion have three parameters (*d*, *h*, *E*). The Young’s modulus of background tissue phantom is 5 kPa, and the height is set at 20 mm. The schematic of three calibration phantoms are shown in Figure 12. 

**Figure 13:**
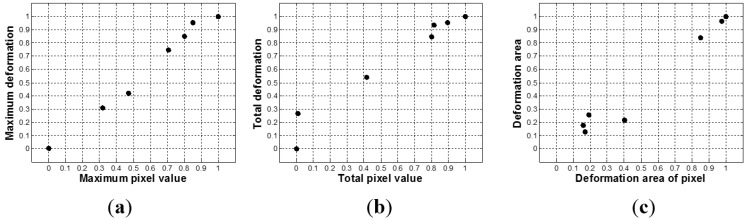
The relationship between FEM tactile data and TSIS tactile data. (**a**) Maximum pixel value *versus* maximum deformation; (**b**) Total pixel value *versus* total deformation, and (**c**) Deformation area of pixel *versus* deformation area.

**Figure 14:**
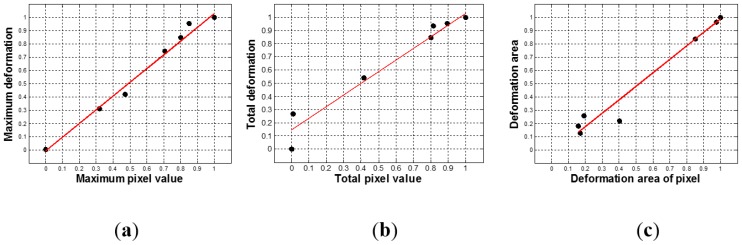
The linear regression results between TSIS tactile data and FEM tactile data. (**a**) The linear regression result between maximum pixel value and maximum deformation; (**b**) The linear regression result between total pixel value and total deformation; and (**c**) The linear regression result between deformation area of pixel and deformation area.

To map the tactile data of TSIS to tactile data of FEM, first, we simulated tactile data of nine inclusions using FEM. The tactile data was then quantified by (*d_total_*, *d_area_*, *d_probe_*). The tactile data on the inclusions in the calibration phantoms was also obtained by the TSIS. The TSIS tactile data is quantified by (*d_total_*, *d_area_*, *d_probe_*). Figure 13 shows the quantified TSIS tactile data results. It can be observed that the results show the similar pattern with the FEM case. To investigate the relationship between FEM tactile data and TSIS tactile data, we generated graphs of ($O_{FEM}^{1}$;$O_{TSIS}^{1}$), ($O_{FEM}^{2}$;$O_{TSIS}^{2}$), and ($O_{FEM}^{3}$;$O_{TSIS}^{3}$) in Figure 14. Linear regression was used to model the relationship between FEM tactile data and TSIS tactile data. 

### 3.5. Inversion Algorithm

**Figure 15:**
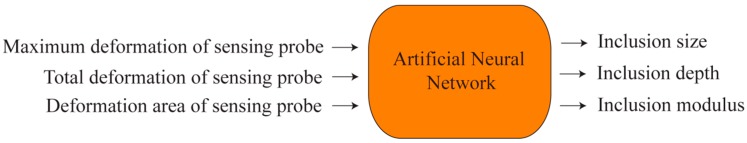
The maximum deformation, total deformation, and deformation area of sensing probe are the inputs of artificial neural network. The outputs of the network are the inclusion size, depth, and Young’s modulus.

The goal of the tactile data processing is to estimate inclusion’s size, depth, and Young's modulus using the tactile data. In the forward modeling, we generated 134 input data sets (*d*, *h*, *E*) and investigated its output data sets (*d_total_*, *d_area_*, *d_probe_*). The 134 data gets were obtained from the FEM simulation. Now, we design the inversion algorithm to estimate (*d*, *h*, *E*) using (*d_total_*, *d_area_*, *d_probe_*). To achieve this purpose, first we need an inversion algorithm training using 134 data sets generated in the forward modeling. In this paper, neural network (NN), is utilized for the inversion algorithm. Figure 15 shows the diagram of inputs and outputs of NN.

**Figure 16:**
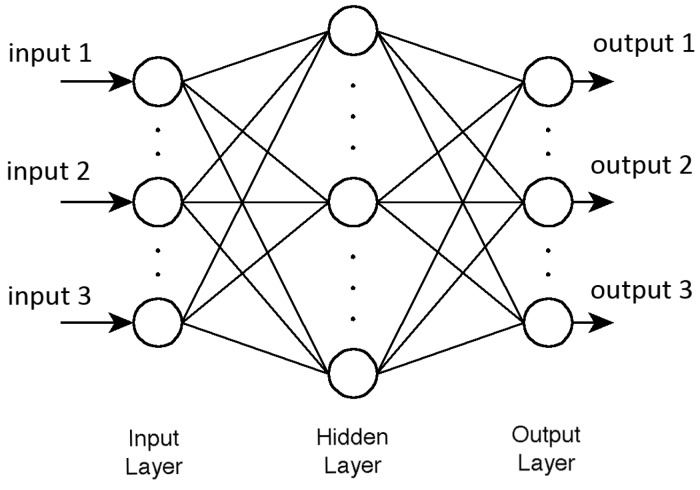
The multilayered neural network structure.

In this study, we have taken multilayered neural network. Multilayered neural network consists of neurons united in layers. Each *i* layer is connected with *i* − 1 and *i* + 1 layers and neurons in layer are not connected to each other. Figure 16 represents the structure of multilayered neural network.

In the structure, too many neurons would lead to over-fitting and great variance of mean squared error (MSE) results, but not enough neurons would cause high MSE results. Thus numbers of neurons and layers were set experimental way to three layers as follows: (1) 1st layer: three neurons, sigmoid activation function; (2) 2nd layer: four neurons, sigmoid activation function; (3) 3rd layer: three neurons, linear activation function.

For training the NN, two different algorithms were considered. The first one is the Levenberg-Marquardt algorithm (LMA) and the second one is the Scaled Conjugate Gradient algorithm (SCGA). The LMA is a very popular curve-fitting algorithm used in many software applications for solving generic curve-fitting problems. The LMA interpolates between the Gauss–Newton algorithm (GNA) and the method of gradient descent. The LMA is more robust than the GNA, which means that in many cases it finds a solution even if it starts very far off the final minimum. On the other hand, for well-behaved functions and reasonable starting parameters, the LMA tends to be a bit slower than the GNA. LMA can also be viewed as Gauss–Newton using a trust region approach. Below is the summary of the LMA.

If a function E(x) is to be minimized with respect to the parameter vector x, then Newton’s method would be:

(1) $$\Delta x=-{\left[\nabla^{2}E(x)\right]}^{-1}\nabla E(x)$$

where $\nabla^{2}E(x)$ is the Hessian matrix and ∇E(x) is the gradient. If E(x) is $E(x)=\sum_{i=1}^{N}e_{i}^{2}(x)$, then it can be shown that:

(2) $$\nabla E(x)=J^{T}(x)e(x)$$

(3) $$\nabla^{2}E(x)=J^{T}(x)J(x)+S(x)$$

where J(x) is the Jacobian matrix and:

(4) $$S(x)=\sum_{i=1}^{N}e_{i}\nabla^{2}e_{i}(x)$$

For the Gauss-Newton method, it is assumed that S(x)≈0. Then Equation (1) becomes

(5) $$\Delta x={\left[J^{T}(x)J(x)\right]}^{-1}J(x)e(x)$$

The Levenberg-Marquardt modification to the Gauss-Newton method is

(6) $$\Delta x={\left[J^{T}(x)J(x)+\mu I\right]}^{-1}J(x)e(x)$$

The parameter μ is multiplied by some factor β whenever a step would result in an increased E(x). When a step reduces E(x), μ is divided by β. When the scalar μ is very large, the LMA approximates the steepest descent method. However, when μ is small, it is the same as the GNA. Since the GNA converges faster and more accurately towards an error minimum, the goal is to shift towards the GNA as quickly as possible. The value of μ is decreased after each step unless the change in error is positive; *i.e.* the error increases. For the NN mapping problem, the terms in the Jacobian matrix can be computed by a simple modification to the back-propagation algorithm.

As alternate to LMA is SCGA. The SCGA is based upon a class of optimization techniques well known in numerical analysis as the Conjugate Gradient Methods. SCGA uses second order information from the NN but requires only O(N) memory usage, where N is the number of weights in the network. A brief description of SCGA goes follows. Let’s take a Taylor expansion of E(x) function:

(7) $$E(x+\Delta x)=E(x)+\nabla E{\left(x\right)}^{T}\Delta x+\frac{1}{2}\Delta x^{T}\nabla^{2}E(x)\Delta x+\ldots$$

The Conjugate Gradient Methods are based on the general optimization strategy, but choose the search direction and the step size more carefully by using information from the second order approximation:

(8) $$E(x+\Delta x)\approx E(x)+\nabla E{\left(x\right)}^{T}\Delta x+\frac{1}{2}\Delta x^{T}\nabla^{2}E(x)\Delta x$$

It is not desirable to calculate the Hessian matrix explicitly, because of the calculation complexity and memory usage involved. The solution is

(9) $$s_{k}=\nabla^{2}E(x_{k})p_{k}\approx \frac{\nabla E(x_{k}+\sigma_{k}p_{k})-\nabla E(x_{k})}{\sigma_{k}}$$

The idea is to introduce a scalar $\lambda_{k}$, which is supposed to regulate the indefiniteness of $\nabla^{2}E(x)$ such as in LMA. This is done by setting as:

(10) $$s_{k}=\frac{\nabla E(x_{k}+\sigma_{k}p_{k})-\nabla E(x_{k})}{\sigma_{k}}+\lambda_{k}p_{k}$$

and for each iteration adjusting $l_{k}$ looking at the sign of $d_{k}$. The summary of the SCGA is as shown below.

***Step 1*:** Choose weight vector $x_{1}$ and scalars σ>0, $\lambda_{k}>0$ and $\bar{\lambda_{k}}=0$. Set $p_{1}$ = $r_{1}$ = $-\nabla E(x_{1})$, k=1, and set *success = true*.

***Step 2*:** If *success = true* then calculate second order information:

$$\begin{array}{l} \sigma_{k}=\frac{\sigma }{\left|p_{k}\right|}\\ s_{k}=\frac{\nabla E(x_{k}+\sigma_{k}p_{k})-\nabla E(x_{k})}{\sigma_{k}}\\ \delta_{k}=p_{k}^{T}s_{k} \end{array}$$

***Step 3*:** Scale $s_{k}$ :

$$\begin{array}{l} s_{k}=s_{k}+(\lambda_{k}-\bar{\lambda_{k}})p_{k}\\ \delta_{k}=\delta_{k}+(\lambda_{k}-\bar{\lambda_{k}}){\left|p_{k}\right|}^{2} \end{array}$$

If $\sigma_{k}\leq 0$ then make the Hessian matrix positive definite:

$$\begin{array}{l} s_{k}=s_{k}+(\lambda_{k}-2\frac{\delta_{k}}{{\left|p_{k}\right|}^{2}})p_{k}\\ \bar{\lambda_{k}}=2\left(\lambda_{k}-2\frac{\delta_{k}}{{\left|p_{k}\right|}^{2}}\right)\\ \delta_{k}=-\delta_{k}+(\lambda_{k}-\bar{\lambda_{k}}){\left|p_{k}\right|}^{2},\;\lambda_{k}=\bar{\lambda_{k}} \end{array}$$

***Step 4*:** Calculate step size:

$$\mu_{k}=p_{k}^{T}r_{k},\;\alpha_{k}=\frac{\mu_{k}}{\delta_{k}}.$$

***Step 5*:** Calculate the comparison parameter: $\Delta_{k}=\frac{2\delta_{k}\left[E(x_{k})-E(x_{k}+\alpha_{k}p_{k})\right]}{\mu_{k}^{2}}.$

***Step 6*:** If $\Delta_{k}\geq 0$ then a successful reduction in error can be made:

$$\begin{array}{l} x_{k+1}=x_{k}+\alpha_{k}p_{k}\\ r_{k+1}=-\nabla E(x_{k+1})\\ \bar{\lambda_{k}}=0,\;succes=true \end{array}$$

***Step 7 a*:** If k mad N = 0 then restart algorithm: pk+1=rk+1

Else create new conjugate direction:

$$\begin{array}{l} \beta_{k}=\frac{{\left|r_{k+1}\right|}^{2}-r_{k+1}r_{k}}{\mu_{k}}\\ p_{k+1}=r_{k+1}+\beta_{k}p_{k} \end{array}$$

***Step 7 b*:** If $\Delta_{k}\geq 0.75$ then reduce the scale parameter: $\lambda_{k}=\frac{1}{2}\lambda_{k}$

Else a reduction in error is not possible: $\bar{\lambda_{k}}=\lambda_{k},\;succes=false$

***Step 8*:** If $\Delta_{k}<0.25$ then increase the scale parameter: $\lambda_{k}=4\lambda_{k}$

***Step 9*:** If the steepest descent direction $r_{k}\neq 0$ then set k=k+1 and go to ***step 2***

Else terminate and return $x_{k+1}$ as the desired minimum.

## 4. Experimental Results

For the validation of the network model, we used two types of cross validation method. 

### 4.1. Validation Method

The first one is the hold out validation (HOV) and the second one is the leave one out cross validation (LOOCV). Below is the description of these two validation methods. Hold-out validation (HOV) is the most common validation method of the NN. In this method, an independent test set is preferred to avoid over-fitting. A natural approach is to split the available data into two non-overlapped parts: one for training and the other for testing. The test data is held out and not looked at during training. HOV avoids the overlap between training data and test data, yielding a more accurate estimate for the generalization performance of the algorithm. The downside is that this procedure does not use all the available data and the results are highly dependent on the choice for the training/test data split. In the current 134 data sets, we use ninepoints data of the calibration phantom as test data. 

Leave-one-out cross-validation (LOOCV) is a special case of *k*-fold cross-validation where *k* equals the number of instances in the data. In other words, in each iteration, nearly all the data except for a single observation are used for training and the network model is tested on the single observation. An accuracy estimate obtained using LOOCV is known to be almost unbiased but it has high variance, leading to unreliable estimates. It is still widely used when the available data are very rare, especially in bioinformatics where only dozens of data samples are available. 

The final estimation error is average of iterations’ estimation error. In this study, the mean squared error (MSE) is used to represent the estimation error. The MSE is computed as follows. Let *P* be input data matrix, *T* be output data matrix and *Y* be network’s output data matrix. Then MSE e is calculated as below.

(11) e=average(sum(T−Y))×100 (%)

where i is the test data number and j is the output number.

### 4.2. Test Results

**Figure 17:**
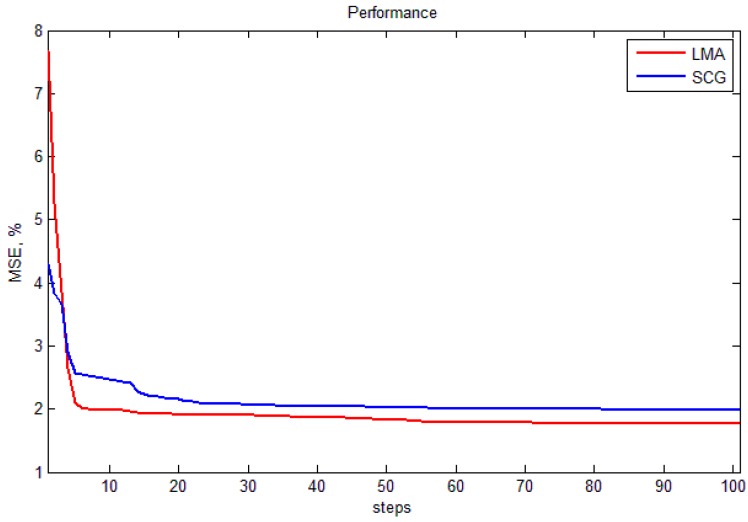
The performance plot of LMA and SCGA.

In this section, two training algorithms, LMA and SCGA, are validated using two validation methods, HOV and LOOCV. Figure 17 shows performance plot of LMA and SCGA. It shows that LMA works with higher accuracy than SCG and there is no need to train network more than 100 iterations. The test results of the NN are averaged with 10, 50, and 100 experiments. Table 1 represents the final validation results. From the results, we notice that both SCGA and LMA’s train results are good; however, LMA seems to be over-fitted already after 100 iterations because of high errors. Best result shows SCG with LOOCV at 100 iterations. The results are 0.58% MSE of size, 3.82% MSE of depth, and 2.51% MSE of hardness. The reason why we used FEM and ANN algorithms is because of its efficiency. In the future work, we will compare these two algorithms with other algorithms and show the performance of the proposed sensor. 

**Table 1:** LMA and SCGA validation using HOV and LOOCV.

	Iterations	Hold-Out Validation						Leave-One-Out Cross Validation					
		10		50		100		10		50		100	
	Output #	Train	Test	Train	Test	Train	Test	Train	Test	Train	Test	Train	Test
SCGA	1 (size)	0.79	1.58	0.3	1.03	0.27	0.99	0.93	1.72	0.32	0.79	0.29	0.58
	2 (depth)	2.7	4.08	2.59	3.99	2.56	4.09	2.78	4.1	2.66	3.87	2.62	3.82
	3 (modulus)	3.16	6.6	3.1	6.58	3.09	4.6	3.36	6.59	3.28	6.64	3.24	6.9
LMA	1 (size)	0.28	1.06	0.26	3.19	0.26	6.84	0.29	0.52	0.26	1.03	0.25	3.08
	2 (depth)	2.54	5.21	2.36	39.1	2.25	105	2.59	3.86	2.42	6.25	2.29	33.8
	3 (modulus)	3.05	6.53	2.91	12.1	2.77	46.4	3.16	6.9	2.83	13.3	2.68	43.1

## 5. Conclusions 

In this paper, an optical based tissue inclusion parameter estimation method is proposed to quantify absolute stiffness and geometric parameters of tissue inclusions. The estimation is performed based on the tactile data obtained by the tactile sensation imaging system. To design the estimation method, we used finite element method based forward algorithm and artificial neural network based inversion algorithm. The performance of the method was experimentally verified using realistic tissue phantoms with embedded stiff inclusions. The experimental results showed that, in the best case, the proposed estimation method can measure the size, depth, and Young's modulus of a tissue inclusion with 0.58%, 3.82%, and 2.51% relative error, respectively. 
